# Anti-cancer drug 3,3′-diindolylmethane activates Wnt4 signaling to enhance gastric cancer cell stemness and tumorigenesis

**DOI:** 10.18632/oncotarget.7684

**Published:** 2016-02-24

**Authors:** Yanhua Zhu, Bin Zhang, Aihua Gong, Hailong Fu, Xu Zhang, Hui Shi, Yaoxiang Sun, Lijun Wu, Zhaoji Pan, Fei Mao, Wei Zhu, Hui Qian, Wenrong Xu

**Affiliations:** ^1^ Key Laboratory of Laboratory Medicine of Jiangsu Province, The Affiliated Hospital, School of Medicine, Jiangsu University, Zhenjiang, Jiangsu, P. R. China; ^2^ Department of Clinical Laboratory, Dali Bai Autonomous Prefecture People's Hospital, Dali, Yunnan, P. R. China

**Keywords:** 3,3′-diindolylmethane, gastric cancer, Wnt4, β-catenin, stemness, tumorigenesis

## Abstract

As a natural health supplement, 3,3′-diindolylmethane (DIM) is proposed as a preventive and chemotherapeutic agent for cancer by inhibiting cell proliferation and inducing cell apoptosis. However, we found that in contrary to high level of DIM (30 μM), low level of DIM (1 μM and 10 μM) obviously promoted gastric cancer cell growth and migration. In addition, we found that low level of DIM increased the expression of stemness factors and enhanced the pluripotency of gastric cancer cells. Low level of DIM promoted gastric cancer progression by inducing the PORCN-dependent secretion of Wnt4 and the activation of β-catenin signaling. Wnt4 knockdown reversed the effects of low level of DIM on gastric cancer cells. The results of *in vivo* studies showed that gastric cancer cells treated with low level of DIM (1 μM) grew faster and expressed higher level of Wnt4 than control cells. Taken together, our findings indicate that low level of DIM activates autocrine Wnt4 signaling to enhance the progression of gastric cancer, which may suggest an adverse aspect of DIM in cancer therapy. Our findings will provide a new aspect for the safety of DIM in its clinical application.

## INTRODUCTION

Gastric cancer is one of the most lethal cancers in Asia and the second leading cause of cancer-related death worldwide [1-3]. Chemotherapy is one of the primary treatments for gastric cancer. Although many new anticancer drugs and chemotherapies have been introduced, there has been no significant progress in the treatment effect. The main reason is that gastric cancer cells can develop multidrug resistance to chemotherapeutic drugs, which significantly limits the application of chemotherapy drugs [4, 5]. The natural indoles DIM is a small molecule compound and an major active metabolite of indole-3-carbinol, which is also a natural health supplement as well as a naturally-occurring compound in cruciferous vegetables such as broccoli, brussels sprouts, and cabbage [6–10]. Many studies have shown that DIM has anticancer effects in various cancer cells *in vitro* and *in vivo*. DIM induces a G1 cell cycle arrest in breast, prostate, colon, and esophageal cancer cells [11–15]. DIM inhibits cell proliferation and induces cell apoptosis in cancer cells [7, 16–18]. Moreover, DIM could suppress tumor growth by inhibiting angiogenesis [19, 20]. Interestingly, low level of DIM could activate estrogen receptor α and induce the proliferation of breast cancer cells in the absence of estradiol [21]. The above mentioned studies indicate that DIM at certain doses could effectively inhibit cancer progression. However, the effects of low level of DIM on cancer remain largely unknown.

Cancer stem cells (CSCs) are known to be resistant to conventional chemotherapy and radiotherapy, which could promote tumor growth through self-renewal and differentiation [3, 22–25]. Recently, many studies have shown that small molecule drugs could promote cell reprogramming and stemness [26–27, 29]. Small molecules have positive impacts on stem cell biology, from the maintenance of pluripotency, the promotion of single cell survival and steering differentiation to involvement in reprogramming somatic cells [26]. Pluripotent stem cells can be generated from mouse somatic cells at a frequency up to 0.2% using a combination of seven small-molecule compounds [27]. The previous study has suggested a new approach to regulate somatic cell reprogramming by targeting chromatin de-condensation with small molecules [28]. Primordial germ cells can be reprogrammed into pluripotent state using small molecule compounds [29]. The chemotherapeutic agents could induce the enrichment of CSCs in several types of cancer. However, the effects of DIM, the small molecule compound, on the stemness of gastric cancer cells are not well characterized.

In this study, we investigated the effects of low level of DIM on gastric cancer cells and explored the underlying molecular mechanisms. We found that low level of DIM (1 μM and 10 μM) could obviously promote gastric cancer cell proliferation, migration, and stemness through the activation of autocrine Wnt4/β-catenin signaling.

## RESULTS

### Low level of DIM promotes the proliferation and migration of gastric cancer cells

**Figure 1:**
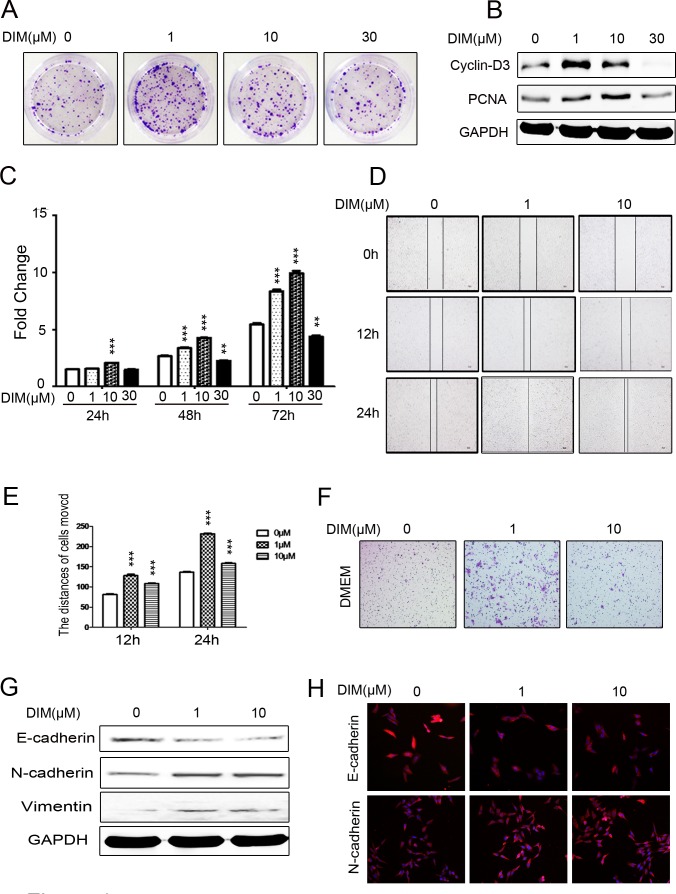
Low level of DIM promotes the proliferation and migration of gastric cancer cells **A.** Representative images of colony formation in HGC-27 cells treated with 0, 1, 10, and 30 μM DIM. Original magnification, 40 Χ. **B.** Western blotting assays for the expression of Cyclin-D3 and PCNA proteins in HGC-27 cells treated with 0, 1, 10, and 30 μM DIM for 48 h. **C.** Cell counting assay for HGC-27 cells treated with 0, 1, 10, and 30 μM DIM for 24, 48 and 72h. **D.** Wound healing assays for the migratory ability of HGC-27 cells at 12 and 24 h after the treatment with DIM (1 and 10 μM). Original magnification, 100Χ. **E.** The gap distance in **A.** was quantified. ***P < 0.001. **F.** The migratory ability of HGC-27 cells treated with 0, 1, and 10 μM DIM was evaluated by using transwell migration assay. Original magnification, 100 Χ. **G.** Western blotting assays for the expression of E-cadherin, N-cadherin, and Vimentin in HGC-27 cells treated with 0, 1, and 10 μM DIM for 48 h. **H.** Representative immunofluorescence images of E-cadherin and N-cadherin expression in HGC-27 cells treated with 0, 1, and 10 μM DIM for 48 h. Original magnification, 200 Χ.

To determine the effects of various levels of DIM on human gastric cancer cell growth, we analyzed the colony formation abilities of six gastric cancer cell lines (including HGC-27, MGC-803, SGC-7901, AGS, MKN-45, and BGC-823) which were treated with different concentrations of DIM (ranging from 1 μM to 30 μM) for 10 days. The results showed that 30 μM DIM significantly inhibited the formation of colonies in all the tested gastric cancer cell lines. In contrary, 1 μM and 10 μM DIM dramatically enhanced the colony formation abilities of all the tested gastric cancer cell lines (Figure 1A, Supplementary Figure 1A, 1C, 1E, 1G, 1J). Additionally, 1 μM and 10 μM DIM increased the expression of cell proliferation related proteins PCNA and Cyclin-D3 (Figure 1B, Supplementary Figure 1B, 1D, 1F, 1H, and 1K). Moreover, the results of cell counting assay showed that treatment with 1 μM and 10 μM DIM promoted the proliferation of HGC-27 cells at 48 h and 72 h after seeding while treatment with 30 μM DIM had the opposite effect (Figure 1C). Taken together, these results suggest that low level of DIM promotes the proliferation of gastric cancer cells.

We next determined the effect of low level of DIM on the migration of gastric cancer cells. The results of wound healing assay showed that 1 μM and 10 μM DIM promoted the migration of HGC-27 cells compared to the control group (Figure 1D, 1E). The transwell migration assay was also used to determine the role of low level of DIM in gastric cancer cell motility. Compared with the control group, 1μM and 10μM DIM markedly increased the number of migrated HGC-27 cells (Figure 1F). The similar results were also obtained in the other gastric cancer cells (Supplementary Figure 2A-2D). The results of western blot showed that treatment with 1 μM and 10 μM DIM inhibited the expression of epithelial cell marker E-cadherin and increased the expression of mesenchymal cell markers N-cadherin and Vimentin in HGC-27 cells (Figure 1G). The results of immunofluorescent staining also confirmed the increase of N-cadherin and the decrease of E-cadherin by 1μM and 10 μM DIM in HGC-27 cells (Figure 1H). In summary, these data suggests that low level of DIM enhances the migratory ability of gastric cancer cells.

### Low level of DIM enhances the stemness of gastric cancer cells

**Figure 2:**
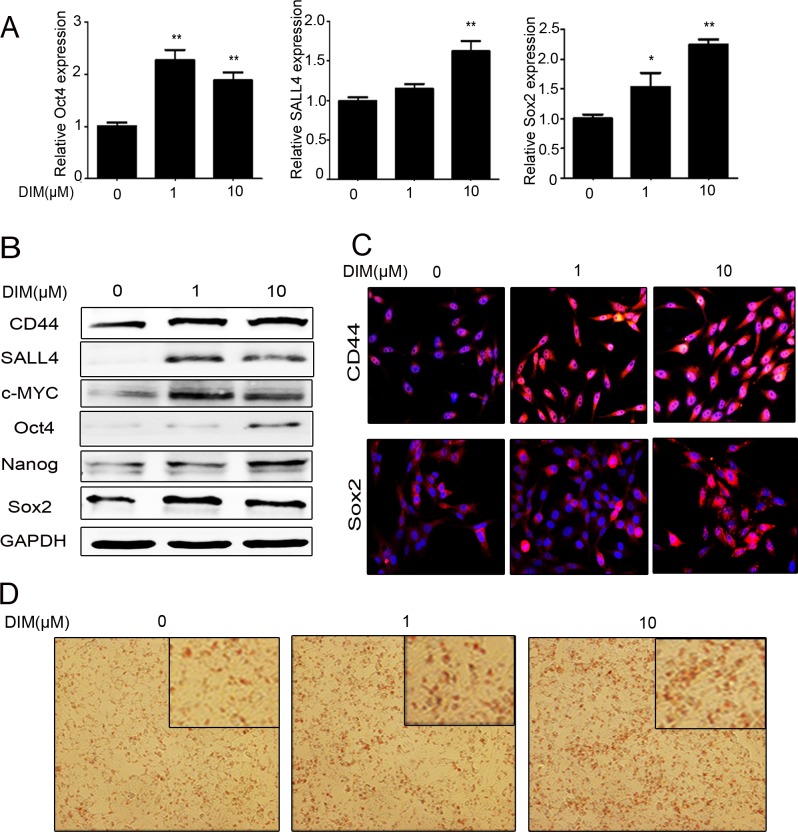
Low level of DIM enhances the stemness of gastric cancer cells **A.** Real-time RT-PCR for the expression of Oct4, SALL4, and Sox2 genes in HGC-27 cells treated with 0, 1, and 10 μM DIM for 48 h. (*n* = 3, *P* < 0.05). **B.** Western blotting assays for the expression of CD44, SALL4, c-MYC, Oct4, Nanog, and Sox2 proteins in HGC-27 cells treated with 0, 1, and 10 μM DIM for 48 h. **C.** Immunofluorescent staining of CD44 and Sox2 proteins in HGC-27 cells treated with 0, 1, and 10 μM DIM for 48 h. Original magnification, 200Χ. **D.** Adipogenic differentiation of HGC-27 cells treated with 0, 1, and 10 μM DIM for 48 h. Original magnification, 200 Χ.

Increasing evidence suggests that cell proliferation and EMT phenotype are closely related to cell stemness [30, 31]. Since low level of DIM obviously enhanced gastric cancer cell growth and migration, we wanted to know whether cell stemness and pluripotency are involved in these changes. We first examined the expression of stem cell markers in HGC-27 cells treated with low level of DIM for 48h. The results of quantitative RT-PCR showed that the expression of Oct4, SALL4, and Sox2 genes was significantly up-regulated in HGC-27 cells treated with 1 μM and 10 μM DIM for 48 h (Figure 2A). The results of western blot showed that the expression of CD44, SALL4, c-MYC, Oct4, Nanog, and Sox2 proteins also increased in 1 μM and 10 μM DIM-treated gastric cancer cells (Figure 2B, Supplementary Figure 3A, 3C, 3E, 3F, 3G). In addition, we confirmed the increased expression of CD44 and Sox2 in HGC-27 cells treated with low level of DIM by using immunofluorescent staining (Figure 2C). We next determined the differentiation potential of HGC-27 cells treated with low level of DIM. The results showed that HGC-27 cells treated with 1 μM and 10 μM DIM could be more efficiently induced to differentiate into adipocytes in the appropriate conditioned medium (Figure 2D). In brief, low level of DIM could enhance the stemness of gastric cancer cells.

### Low level of DIM activates Wnt/β-catenin signaling to promote gastric cancer progression

**Figure 3:**
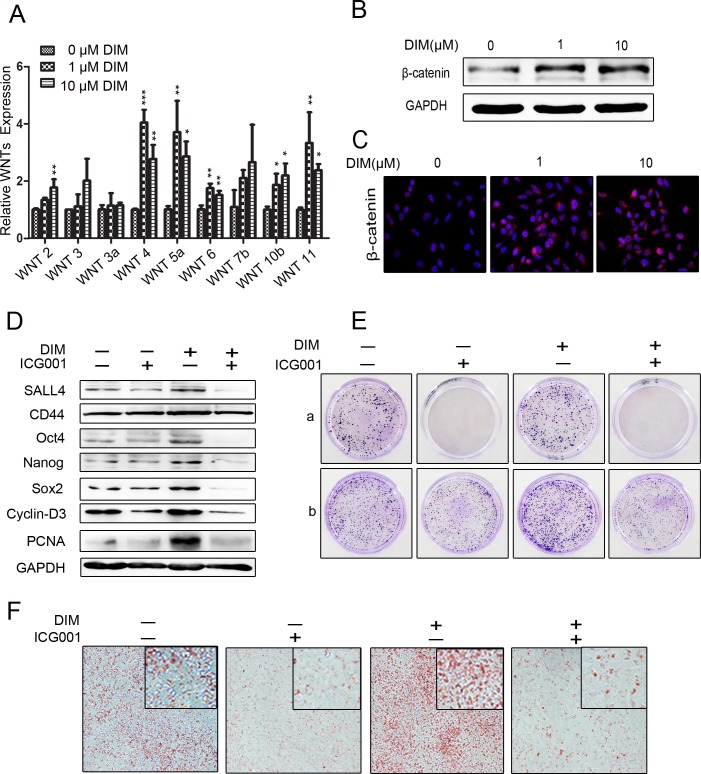
Low level of DIM activates β-catenin signaling to promote gastric cancer progression **A.** Real-time RT-PCR for the expression of WNT2, WNT3, WNT3a, WNT4, WNT5a, WNT6, WNT7b, WNT10b, and WNT11 genes in HGC-27 cells treated with 0, 1, and 10 μM DIM for 48 h. (*n* = 3, *P* < 0.05). **B.** Western blotting assays for β-catenin expression in HGC-27 cells treated with 0, 1, and 10 μM DIM for 48 h. **C.** HGC-27 cells were treated with 0, 1, and 10 μM DIM for 48 h. The nuclear translocation of β-catenin was determined by using immunofluorescence staining. Original magnification, 200Χ. **D.** Western blotting assays for the expression of SALL4, CD44, Oct4, Nanog, Sox2, Cyclin-D3, and PCNA proteins in HGC-27 cells treated with 1 μM DIM in the presence or absence of ICG001 (10 μM/ml) for 48 h. **E.** Representative images of cell colonies in HGC-27 cells treated with 1 μM DIM in the presence or absence of ICG001 (10 μM/ml). **A.** HGC-27 were treated with 1 μM DIM and 10 μM ICG001 for 7 days. **B.** HGC-27 cells were treated with 1 μM DIM and 10 μM ICG001 for 48 h, and then collected for colony formation assay for 7 days. **F.** Adipogenic differentiation of HGC-27 cells treated with 1 μM DIM in the presence or absence of ICG001 (10 μM/ml) for 48 h. Original magnification, 200Χ.

The previous studies indicate that the Wnt/β-catenin pathway is a key regulator of cell survival, proliferation, migration, and stemness [3, 32–34]. We found that low level of DIM induced the expression of WNT2, WNT4, WNT5a, WNT6, WNT10b, and WNT11 in HGC-27 cells. We focused on WNT4 in the following studies as it showed the most significant change (Figure 3A). To test whether Wnt/β-catenin signaling is involved in the effects of low level of DIM on gastric cancer cells, we detected the protein levels of β-catenin in HGC-27 cells treated with low level of DIM by using western blot. We found that the expression of β-catenin and its downstream targets CD44, c-MYC, and Cyclin-D3 were significantly up-regulated in gastric cancer cells after treatment with low level of DIM (Figure 3B). The activation of β-catenin by low level of DIM was also confirmed in MGC-803 and SGC-7901 cells (Supplementary Figure 3B and 3D). The results of immunofluorescent staining showed that low level of DIM obviously induced nuclear translocation of β-catenin in HGC-27 cells (Figure 3C). ICG001, an inhibitor of β-catenin, remarkably reversed the up-regulation of CD44, c-MYC, Nanog, Sox2, PCNA, SALL4, Oct4, and Cyclin-D3 induced by low level of DIM (Figure 3D). In addition, we found that low level of DIM-induced colony formation and adipogenic differentiation of gastric cancer cells were also reversed by ICG001 (Figure 3E and 3F). Taken together, these results suggest that low level of DIM promotes gastric cancer progression through Wnt/β-catenin signaling.

### Low level of DIM promotes Wnts autocrine to activate β-catenin pathway

**Figure 4:**
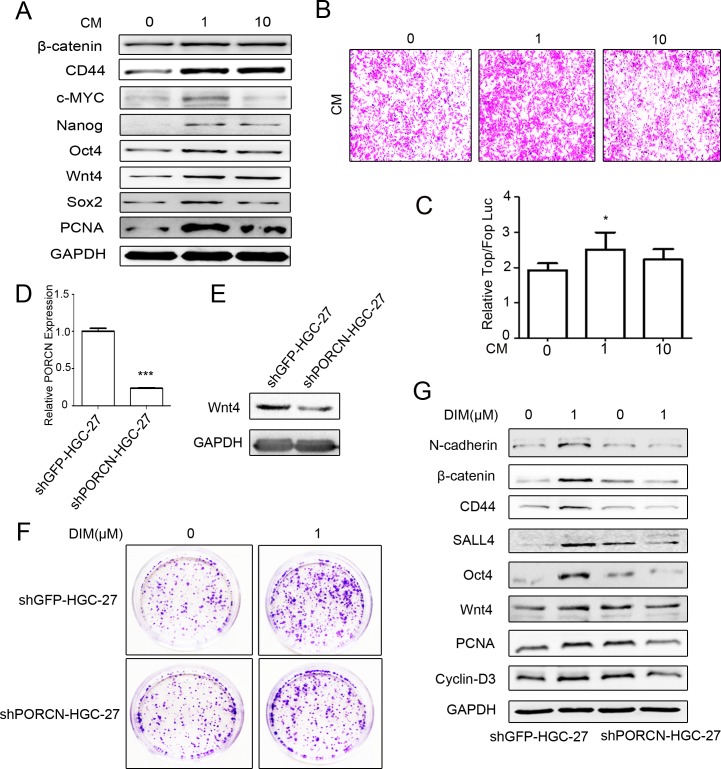
Low level of DIM activates β-catenin signaling by inducing Wnt autocrine **A.** Western blotting assays for the expression of β-catenin, CD44, c-MYC, Nanog, Oct4, Wnt4, Sox2, and PCNA proteins in HGC-27 cells incubated with the conditioned medium form DIM-treated HGC-27 cells for 48 h. **B.** The conditioned medium in the lower chamber of the transwell was collected from HGC-27 cells treated with 0, 1, and 10 μM DIM for 48 h. Original magnification, 100Χ. **C.** HEK293T cells transfected with the TOP-Flash or FOP-Flash luciferase reporter were incubated with equal amounts of conditioned medium collected form HGC-27 cells that were treated with indicated concentrations of DIM for 48 h. The ratio between TOP & FOP Flash luciferase activity was determined at 24h after treatment (*n* = 3, *P < 0.05). **D.** HGC-27 cells were transfected with PORCN-shRNA (shPORCN-HGC-27) or GFP-shRNA (shGFP-HGC-27) lentivirus. The expression of PORCN in HGC-27 cells was determined by real-time RT-PCR. (*n* = 3, ***P < 0.001). **E.** Western blotting assays for Wnt4 expression in shGFP-HGC-27 and shPORCN-HGC-27 cells. **F.** Representative images of colony formation in shGFP-HGC-27 and shPORCN-HGC-27 cells treated with 0 and 1μM DIM. Original magnification, 40Χ. **G.** Western blotting assays for the expression of N-cadherin, β-catenin, CD44, SALL4, Oct4, Wnt4, and PCNA proteins in shGFP-HGC-27 and shPORCN-HGC-27 cells treated with 0 or 1 μM DIM for 48 h.

Wnt ligands activate β-catenin through extracellular receptor [35]. However, whether low level of DIM activates β-catenin by enhancing autocrine Wnts is unclear. To answer this question, we collected the conditioned medium from low level of DIM-treated HGC-27 cells. The results of western blot showed that the conditioned medium promoted the expression of Wnt4, β-catenin, its downstream targets CD44, c-MYC, Cyclin-D3, and transcription factors SALL4, Oct4, Nanog, and Sox2, in HGC-27 cells (Figure 4A). As shown in Figure 4B, the number of migrated HGC-27 cells increased after incubation with the conditioned medium from low level of DIM-treated HGC-27 cells. We further analyzed the activation of β-catenin by using TOP/FOP flash reporter assay. We found that low level of DIM enhanced TOP/FOP flash luciferase reporter activity in HEK293T cells (Figure 4C). The disruption of PORCN impairs the processing and secretion of Wnt proteins [36–38]. Compared to GFP shRNA, PORCN shRNA significantly inhibited the expression of PORCN in HGC-27 cells (Figure 4D). The expression of Wnt4 was also decreased in shPORCN-HGC-27 cells (Figure 4E). The results of colony formation assay showed that low level of DIM accelerated the growth of shGFP-HGC-27 cells while this change was not observed in shPORCN-HGC-27 cells (Figure 4F). The results of western blot showed that the up-regulation of PCNA, N-cadherin, stemness factors (SALL4, Oct4, Nanog, Sox2), Wnt4, β-catenin, and its downstream targets (CD44, c-MYC, and Cyclin-D3) by 1 μM DIM was reversed by PORCN knockdown (Figure 4G). Moreover, the results of immunofluorescent staining confirmed that 1 μM DIM treatment obviously increased the expression of CD44 in shGFP-HGC-27 cells but not in shPORCN-HGC-27 cells (Supplementary Figure 4A). We also observed that 1 μM DIM efficiently induced shGFP-HGC-27 cells to differentiate into adipocytes in the appropriate conditioned medium while had minimal effect on the differentiation potential of HGC-27 cells with PORCN knockdown (Supplementary Figure 4B). In summary, these results indicate that low level of DIM activates β-catenin signaling to promote gastric cancer progression via the autocrine of Wnt.

### Low level of DIM enhances Wnt4 secretion to promote gastric cancer progression

**Figure 5:**
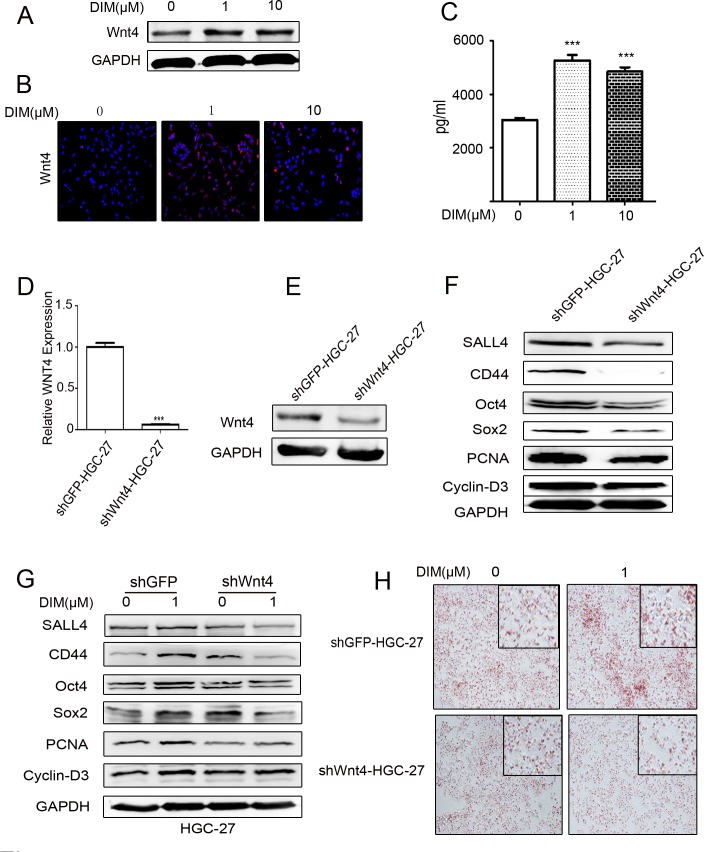
Low level of DIM enhances Wnt4 autocrine to activate β-catenin signaling **A.** Western blotting assays for Wnt4 expression in HGC-27 cells treated with 0, 1, and 10 μM DIM for 48 h. **B.** Immunofluorescence analyses of the expression of Wnt4 in HGC-27 cells treated with 0, 1, and 10 μM DIM for 48 h. Original magnification, 200Χ. **C.** ELISA assays for Wnt4 concentration in the conditioned medium from HGC-27 cells treated with low level of DIM for 48 h (*n* = 3, ***P < 0.001). **D.** HGC-27 were transfected with WNT4-shRNA (shWNT4-HGC-27) or GFP-shRNA(shGFP-HGC-27) lentivirus, respectively. The expression of WNT4 in HGC-27 cells was determined by using real-time RT-PCR. (*n* = 3, ***P < 0.001). **E.** Western blotting assays for Wnt4 expression in shGFP-HGC-27 and shWNT4-HGC-27 cells. **F.** Western blotting assays for the expression of SALL4, CD44, Oct4, Sox2, PCNA, and Cyclin-D3 proteins in shGFP-HGC-27 and shWNT4-HGC-27 cells. **G.** Western blotting assays for the expression of SALL4, CD44, Oct4, Sox2, PCNA, and Cyclin-D3 proteins in shGFP-HGC-27 and shWNT4-HGC-27 cells treated with or without 1μM DIM for 48 h. **H.** Adipogenic differentiation of shGFP-HGC-27 and shWNT4-HGC-27 cells treated with 0 and 1μM DIM for 48 h. Original magnification, 200Χ.

We next wanted to know whether the induction of Wnt4 by low level of DIM is critical for the activation of β-catenin in HGC-27 cells. We found that the expression of Wnt4 protein was increased in the gastric cancer cells treated with low level of DIM by using western blot (Figure 5A, Supplementary Figure 3B and 3D) and immunofluorescent staining (Figure 5B). To confirm these results, we collected the conditioned medium from HGC-27 cells treated with low level of DIM and detected Wnt4 protein levels by using ELISA. The results showed that the expression of Wnt4 was higher in HGC-27 cells treated with low level of DIM for 48 h than that in control cells (Figure 5C). We knocked down the expression of WNT4 in HGC-27 cells by using shRNA (shWNT4-HGC-27) (Figure 5D and 5E). The expression of PCNA, Cyclin-D3, CD44, SALL4, Oct4, Nanog, and Sox2 was decreased in shWNT4-HGC-27 cells (Figure 5F). The induced expression of PCNA, Cyclin-D3, SALL4, CD44, Oct4, and Sox2 in HGC-27 cells by low level of DIM was reversed by WNT4 knockdown (Figure 5G). The knockdown of WNT4 also inhibited the induced differentiation ability of HGC-27 by low level of DIM (Figure 5H). Taken together, these results suggest that low level of DIM promotes gastric cancer progression via Wnt4 autocrine.

### Low level of DIM promotes gastric tumorigenesis *in vivo*

**Figure 6:**
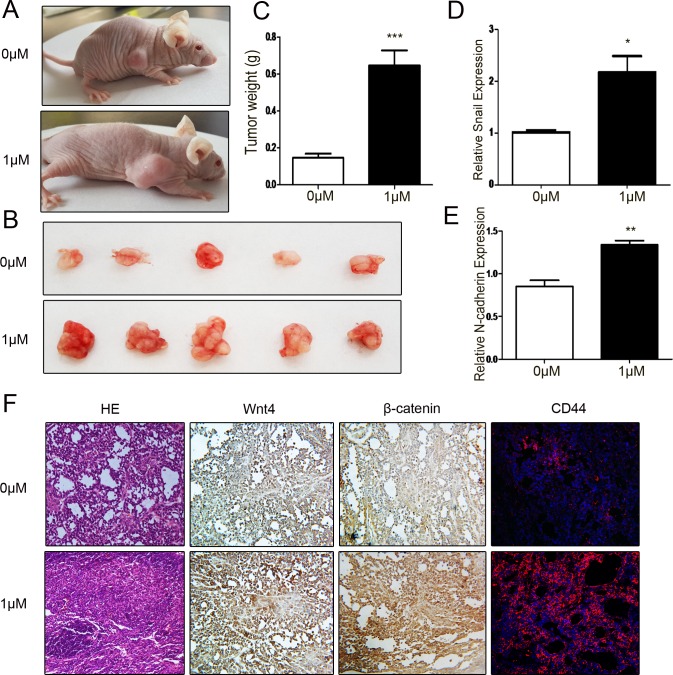
Low level of DIM promotes gastric cancer growth *in vivo* **A.** Representative images of tumor-bearing mice. **B.** The photographs of excised tumors at 28 days post-inoculation. **C.** Tumor weight was evaluated in mice transplanted with SGC-7901 cell that were treated with 0 or 1 μM DIM for 48 h (*n* = 5, ***P < 0.001). **D.** Real-time RT-PCR analyses of Snail and N-cadherin mRNA expression in the xenograft tumors. *P < 0.05, **P < 0.01. **F.** The subcutaneous tumors derived from SGC-7901 cells treated with 0 (upper) and 1 μM DIM (lower) was subjected to H&E staining, immunohistochemical staining of β-catenin and Wnt4, and immunofluorescent analyses of CD44 expression. Original magnification, 200×.

To confirm the *in vitro* results, SGC-7901 cells treated with or without 1 μM DIM were used to establish mouse xenograft tumor models. Representative images of the tumor bearing mice were shown in Figure 6A. The tumors in 1 μM DIM treatment group grew faster and their weight was higher than that in control group (Figure 6B and 6C). The similar results were also observed in MGC-803 cells (Supplementary Figure 5A-5C). Compared with the control group, the expression of Snail and N-cadherin was increased in tumors of 1 μM DIM treatment group (Figure 6D and 6E). The expression of Wnt4 and β-catenin in tumor tissues was determined by using immunohistochemistry. We found that Wnt4 and β-catenin protein levels were higher in 1 μM DIM group than that in control group (Figure 6F). The increased expression of PCNA, Wnt4, β-catenin, and CD44 in 1μM DIM group was also observed (Supplementary Figure 5D). Taken together, these results suggest that low level of DIM enhances gastric cancer growth *in vivo*.

## DISCUSSION

DIM has been proposed as a cancer preventive agent that can be given safely in oral form in repeated doses to rodents and humans [6, 39–40]. The recent reports have shown that DIM could inhibit cell proliferation and induce cell apoptosis in many cancers. DIM inhibits cell growth and induces apoptosis in prostate cancer partially through the regulation of Akt/FOXO3a/GSK-3/β-catenin/AR signaling axis [16, 17]. Several reports have also shown that DIM reduces the activity of nuclear factor-κB (NF-κB) in the prostate and breast cancer cells [41, 42]. DIM suppresses the growth of gastric cancer cells via activation of the Hippo signaling pathway [18]. DIM could strongly inhibit angiogenesis and the growth of transplantable human breast carcinoma in athymic mice [19]. In these studies, a relatively high level of DIM was used. In our work, we explored the effect of low level of DIM on gastric cancer cells and the underlying mechanism. We found that different concentrations of DIM could lead to opposite biological outcomes in gastric cancer. Although 30 μM DIM evidently inhibited gastric cancer cell growth, 1 μM and 10 μM DIM obviously promoted gastric cancer cell proliferation and migration.

Small molecules are more permeable, non-immunogenic, cost-effective, more easily synthesized, preserved, and standardized. Moreover, their effects on inhibiting and activating the function of specific proteins are often reversible and can be finely tuned by varying the concentrations [27]. Small molecule compounds have been found extremely useful to improve the generation of iPSCs [28]. A combination of small molecules can promote the reprogramming of mouse somatic cells into pluripotent stem-like cells [27, 43]. Somatic cells can be reprogrammed to induced pluripotent stem cells (iPSCs) by overexpression of four transcription factors, Oct4, Klf4, Sox2, and c-MYC [44]. In our study, the expression of transcription factors Oct4, Sox2, c-MYC, Nanog and SALL4 as well as the ability to differentiate into adipocytes in gastric cancer cells were enhanced by low level of DIM, suggesting that low level of DIM may affect gastric cancer cell reprogramming.

The Wnt/β-catenin pathway controls embryogenesis and its deregulation is related to tumorigenesis [45–47]. The Wnt/β-catenin pathway critically regulates cell proliferation and apoptosis [3, 32–34]. We found that low level of DIM activated Wnt/β-catenin signaling to enhance gastric cancer cell proliferation and migration. The Wnt/β-catenin signaling is one of the key oncogenic pathways in cancer and targeting this pathway is an attractive therapeutic approach [3, 32]. PORCN is a membrane-bound O-acyltransferase that is required for palmitoylation of Wnt ligands, a necessary step in the processing of Wnt ligand secretion [36–38]. Liu *et al.* suggest that targeting PORCN potently inhibits Wnt signaling [36]. Moreover, PORCN dysfunction produces an “all-WNT” mutant phenotype [37]. These findings provide evidence that PORCN is extremely necessary to Wnts autorcine. The interference with PORCN expression reversed low level of DIM-induced gastric cancer progression, indicating that the autorcine of active Wnt is essential for this process.

Wnt4 is a member of gene family encoding secreted signal protein that participate in carcinogenesis [48]. The up-regulation of Wnt4 is observed in gastric cancer [49]. Wnt4 regulates the proliferation of breast cancer stem cells in response to progesterone [48]. We found that the expression of Wnt4 was obviously increased in gastric cancer cells treated with low level of DIM. The promoting effects of low level of DIM on gastric cancer cells are dependent on Wnt4 secretion. Clevers *et al.* suggest that Wnt molecules transmit downstream signals mainly through extracellular receptor [35]. Luga *et al.* demonstrate that fibroblast-secreted exosomes play a key role in promoting breast cancer cell motility and metastasis by mobilizing autocrine Wnt-planar cell polarity signaling [50]. The SP cells in lymphoma export Wnt3a via exosomes to neighboring cells, thus modulating population equilibrium [34]*.* We have previously shown that exosomes deliver Wnt4 protein from stem cells to skin cells [51, 52], indicating that the low level of DIM-induced Wnt4 protein may be released in exosome form.

The findings of our study may have several implications for the use of DIM as a dietary supplement or as a therapeutic agent. As the blood concentrations of DIM are different in individual patients, it is therefore needed to determine the pharmacokinetic background of the patients in metabolizing DIM to ensure therapeutic safety. In addition, since the low levels of DIM are more achievable than high levels during treatment, the use of DIM should be undertaken very cautiously. Moreover, as demonstrated in this study, the activation of Wnt4 signaling is required by low level of DIM in promoting gastric cancer progression, probably simultaneous targeting Wnt4 may help improve therapeutic efficacy.

In summary, we demonstrate in this study that low level of DIM promotes gastric cancer progression through Wnt4 autocrine and the activation of β-catenin pathway. Our findings not only provide new evidence for gastric cancer progression driven by Wnt/β-catenin signaling but also suggest the adverse aspect of DIM in cancer therapy.

## MATERIALS AND METHODS

The study was approved by the ethical committee of Jiangsu University (2012258).

### Cell lines and cell culture

Human gastric cancer cell lines HGC-27, SGC-7901, and MGC-803 were purchased from the Institute of Biochemistry and Cell Biology at the Chinese Academy of Sciences (Shanghai, China). HGC-27, SGC-7901, and MGC-803 cells were propagated in high-glucose DMEM (Gibco, Grand Island, USA). All the media were supplemented with 10% fetal bovine serum (FBS; Gibco). Cells were cultured at 37°Cin humidified air with 5% CO_2_.

### Colony formation assay

Cells were harvested and seeded into 35-mm plates (1000 cells/well) overnight under standard conditions. Cells were then treated with various doses of DIM (0μM, 1μM, 10μM, 30μM) and incubated at 37°C in a 5% CO_2_ humidified incubator for 10 days. The medium containing various levels of DIM was changed at 3-days interval. At the end of the incubation period, the cultures were fixed with 4% paraformaldehyde and stained with crystal violet.

### Cell counting

Cells were seeded into 24-well plate (4000 cells/well). After 24 h of incubation under standard conditions, the cells were treated with the indicated doses of DIM (0μM, 1μM, 10μM, 30μM). The cells were collected and counted at the indicated time points (24 h, 48 h, 72 h). Fold change of cell number was calculated.

### Transwell migration assay

Cells (1Χ10^5^/well) were plated into the top chamber and 10% FBS containing medium was placed into the bottom chamber. After incubation at 37°C in 5% CO_2_ for 12 h, the cells remaining at the upper surface of the membrane were removed with a cotton swab. The cells that migrated through the 8-μm sized pores and adhered to the lower surface of the membrane were fixed with 4% paraformaldehyde, stained with crystal violet and photographed.

### Wound healing assay

Cell motility was assessed by an *in vitro* wound healing assay. 2Χ10^5^ cells were seeded into six-well plate and cultured in DMEM to reach confluence. A scrape was made in the center of the cell monolayers with a sterile pipette tip to create a gap of constant width. Cellular debris was removed by gently washing with PBS. The initial images of the wounds were captured under phase contrast microscopy and the wounded monolayers were incubated further at 12 h and 24 h after the treatment with fresh DMEM medium in the presence of DMSO or DIM (1 μM or 10 μM). Pictures were taken with a Nikon eclipse Ti-S microscope at a 100Χ magnification. To quantify the cell migration index, photographs of the initial wounded monolayers were compared with the corresponding images of cells at the end of the incubation. Artificial lines fitting the cutting edges were drawn on pictures of the original wounds and overlaid on the images of cultures after incubation. Cells that migrated across the lines that were the distance of cells moved were counted. All quantification was done on full-size images with the weight of artificial lines negligible, when compared with the size of the cell body. At least five fields from each triplicate treatment were counted.

### Western blot

Gastric cancer cells were homogenized and lysed in RIPA buffer supplemented with proteinase inhibitors. Equal amount of proteins were loaded and separated on a 12% SDS-PAGE (sodium dodecyl sulfate-polyacrylamide gel electrophoresis) gel. Following electrophoresis, the proteins were transferred to a PVDF (polyvinylidene difluoride) membrane, blocked in 5% (w/v) non-fat milk and incubated with the primary antibodies. The sources of primary antibodies were: β-catenin and E-cadherin (Cell Signaling Technology, Beverly, MA, USA); SALL4 and N-cadherin (Abcam, USA); Sox2 (Milipore, USA); Wnt4 (Santa Cruz Biotechnology, Santa Cruz, CA, USA); CD44, PCNA, and Cyclin D3 (Bioworld Technology, Louis Park, MN, USA); Oct4 and Nanog (SAB, USA); Vimentin (Signalway Antibody, USA); GAPDH, Goat anti rabbit IgG (HRP), and Goat anti mouse IgG(HRP) (CWBIO).

### RNA extraction, RT-PCR and real-time RT-PCR

Total RNA was extracted from cells and tissues using TRIZOL Reagent (Invitrogen, Carlsbad, CA, USA) according to the manufacturer's instructions, and equal amount of RNA was used for RT-PCR and real-time RT-PCR analyses. β-actin was used as the internal control. The sequences of specific primers are listed in Supplementary Table 1.

### Immunofluorescence

Cells cultured in 24-well chamber slides were treated for 48 h with various concentrations of DIM and then washed twice with cold phosphate-buffered saline (PBS), fixed with 4% paraformaldehyde for 20 min, permeabilized with 0.1% Triton Χ-100 for 10 min, blocked with 5% BSA, incubated with the indicated primary antibodies at 4°C overnight and followed by a Cy3-conjugated anti-rabbit (CD44, Sox2, Wnt4, β-catenin, and E-cadherin) or anti-mouse (N-cadherin) secondary antibody. The cells were then stained with Hoechst33342 for nuclear staining for 5 min, and the images were acquired with a Nikon eclipse Ti-S microscope.

### Adipogenic differentiation

1Χ10^5^ cells were seeded in six-well plate and cultured in DMEM with 10% FBS for 24h and transferred to the medium with different concentrations of DIM for 48 h. The cells were then changed to adipogenic differentiation medium for 1 week according to the manufacturer's instructions (GUXMX-90031, Cyagen Biosciences, CA, USA). At the end of induction, the adipogenic potential was identified by oil red O staining.

### Wnt reporter activity assay

For the luciferase reporter assay, HEK293T cells were co-transfected with TOP-Flash or FOP-Flash luciferase reporter. Transfection efficiency was normalized by cotransfection with a β-actin Renilla reporter containing a Renilla luciferase gene under the control of a human β-actin promoter. The activities of firefly luciferase and Renilla luciferase were quantified by the dual luciferase reporter assay system (Promega). At 6 hours after transfection, HEK293T cells were incubated with equal amounts of conditioned medium from HGC-27 cells treated with indicated concentration of DIM for 48 h. Wnt reporter activity was determined by dual luciferase reporter assay.

### ELISA for Wnt4 detection

HGC-27 cells were treated with low level of DIM for 48 h, then washed with PBS twice, cultivated in the normal high-glucose DMEM culture medium for 48 h, finally the conditioned medium were collected from HGC-27 cells. Wnt4 concentration in cell culture supernatant was measured by using a commercial ELISA Kit according to the manufacturer's instruction(Santa Cruz Biotechnology, Santa Cruz, CA, USA). The absolute amount of Wnt4 protein was calculated based on standard curves using human recombinant Wnt4 as the standard material. The concentration of Wnt4 was expressed as pictograms per milliliter.

### Lentiviral knockdown of PORCN and WNT4 in HGC-27

The lentiviral expression vector containing the PORCN or WNT4 shRNA sequence (sigma) was selected for specifically targeting PORCN and WNT4, which was classified as LentiPORCN-shRNA, LentiWNT4-shRNA, and LentiGFP-shRNA as negative control vector. The PORCN and WNT4 shRNA lentivirus vectors were generated by ligating the vector Tet-pLKO-puro. PORCN shRNA oligonucleotide sequences are: Forward,5′-CCGGCCTTCCACTTCAGCAACTATTCTCGAGAAT AGTTGCTGAAGTGGAAGGTTTTTTG - 3′; Reverse,5′-AATTCAAAAACCTTCCACTTCAGCAACTATTCTCGAGAATAGTTGCTGAAGTGGAAGG - 5′.WNT4 shRNA oligonucleotide sequences are: Forward,5′-CCGGCCCAAGAGATACTGGTTGTATCTCGAGATACAACCAGTATCTCTTGGGTTTTTG - 3′; Reverse, 5′ -AATTCAAAAA CCCAAGAGA TACTGG TTGT ATCTCGAGATACAACCAGTATCTCTTGGG - 3′. The sequences of control shRNA are: Forward, 5′-CCGGGCAAGCTGAGGTCACGTTGCTTTTTG-3′ Reverse, 5′ - AATTCA A AAA GCAAGCTGACCCTGAAGTTCATCT CGAGATGAAC TTCAGGGTCACGTTGC - 3′. The recombinant lentivirus was produced by co-transfecting HEK293T cells with PLKO-GFP-shRNA or PLKO PORCN-shRNA, PLKO-WNT4-shRNA, PU1562 and PU1563 plasmid by using Lipofectamine 2000 (Invitrogen). The virus-containing supernatant was harvested at 48h and 72h post-transfection. HGC-27 cells were transduced with the prepared lentivirus (LentiWNT4-shRNA or Lenti-GFP-shRNA). Stable cell lines were obtained after selection with 1 μg/mL of puromycin (Invitrogen) for 15 days. The expression of shRNA was induced by addition of 80 μg/mL doxycycline. The efficiency of WNT4 knockdown was evaluated by using real-time quantitative RT-PCR and western blot.

### Animal model

Ten male BALB/c nu/nu mice (Laboratory Animal Center of Shanghai, Academy of Science, Shanghai, China) aged 4-6 weeks were randomly divided into two groups (five mice/group). SGC-7901 cells were treated with or without 1 μM DIM for 48 h *in vitro*, then 2.5Χ10^6^ of the cells in 200 μL PBS were implanted subcutaneously into the right flanks of the mice. The nude mice were feed normally and the tumors were harvested at 28 days after the implantation. The tumor size and weight were measured. For MGC-803 cells, the tumors were excised at 30 days post-inoculation.

### Histology and immunohistochemistry

Animals were sacrificed approximately 2 weeks after tumor formation, and the tumors were harvested. Hematoxylin-eosin (H&E) staining and immunohistochemistry were performed on 5μm serial coronal sections from paraffin-embedded tumors. Tissue sections were de-waxed in xylene and rehydrated in graded alcohol, after which endogenous peroxidase activity was quenched by incubating the sections in 0.3% (v/v) hydrogen peroxide in methanol. Antigen-retrieval was performed by incubating the sections in citrate buffer (pH 6.0). Non-specific binding was blocked by 5% bovine serum albumin, and tissue sections were incubated with antibodies against human Wnt4 and β-catenin according to the manufacturer's instructions (Boster Bioengineering Company Limited, Wuhan, China). Anti-Wnt4 (Santa Cruz Biotechnology, Santa Cruz, CA, USA) 1:50; anti-β-catenin (Cell Signaling Technology, Beverly, MA, USA) 1:50; were used as primary antibody. Finally, the slices were mounted with neutral gum for microscopic examination, and cells with brown granules in the cytoplasm or nucleolus were considered positive. Images were collected using a Nikon eclipse Ti-S microscope at a 200Χ magnification.

### Statistical analysis

All data were shown as means ± standard deviation (SD). The statistically significant differences between groups were assessed by analysis of variance (ANOVA) or *t*-test using Prism software (GraphPad, San Diego, USA). *P* value <0.05 was considered significant.

## SUPPLEMENTARY MATERIALS FIGURES AND TABLE


