# Supplementary material for: Anti-cancer drug 3,3′-diindolylmethane activates Wnt4 signaling to enhance gastric cancer cell stemness and tumorigenesis
Source: Oncotarget. 2016 Feb 24;7(13):16311–24. doi: 10.18632/oncotarget.7684 (PMC4941316; doi:10.18632/oncotarget.7684)
Supplement: Supplementary file 1 [file oncotarget-07-16311-s001.pdf]

Anti-cancer drug 3,3′-diindolylmethane activates Wnt4 signaling to enhance gastric cancer cell stemness and tumorigenesis

Supplementary materials

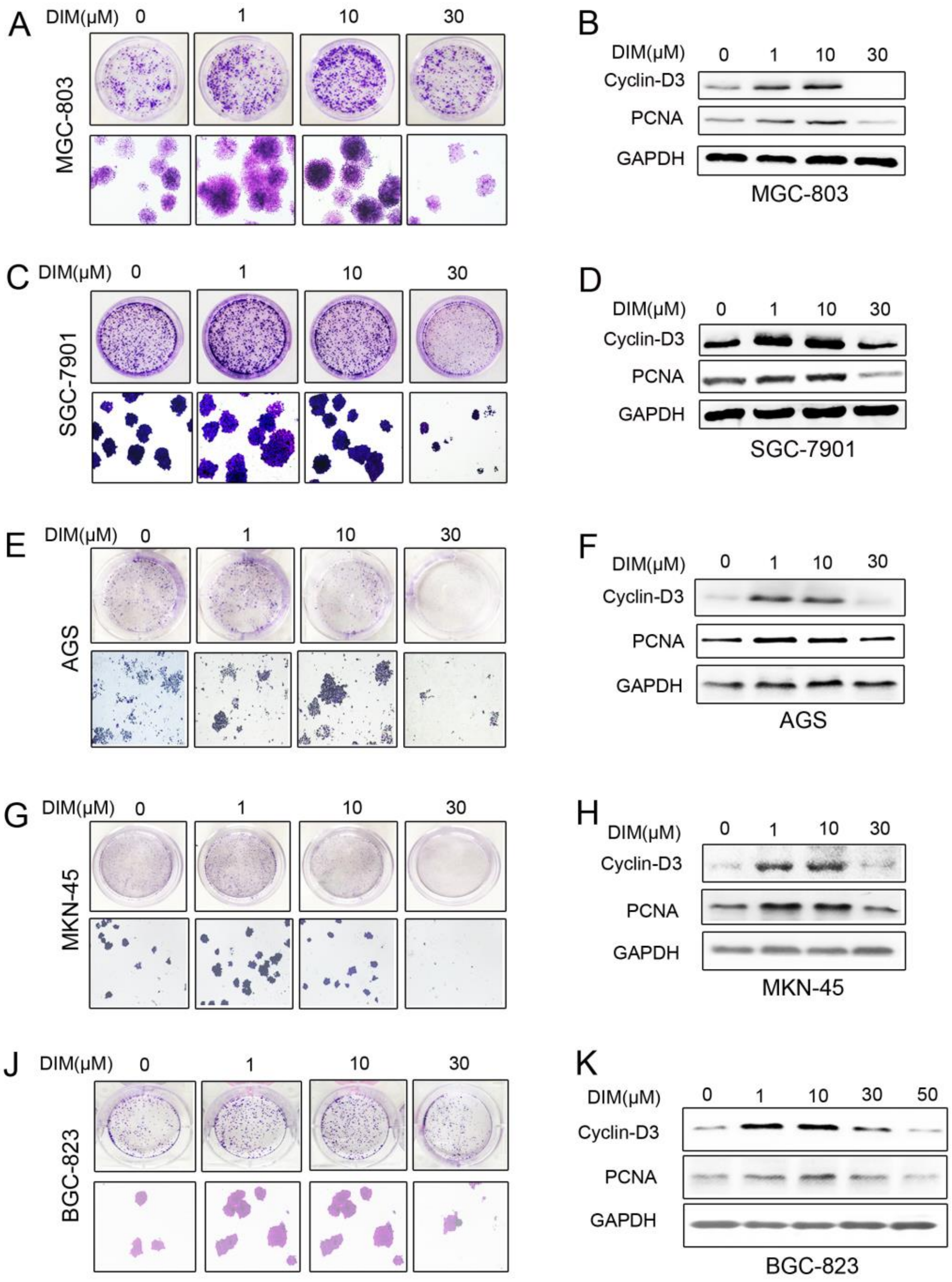

Supplementary Figure 1. **Low level of DIM promoted gastric cancer cells proliferation**

(A, C, E, G, J) Representative images of colony formation in MGC-803, SGC-7901,

AGS, MKN-45 and BGC-823 cells treated with 0μM, 1μM , 10μM and 30μM DIM. Original magnification, 40X. (B, D, F, H, K) Western blot assay for the expression of Cyclin-D3 and PCNA proteins in MGC-803, SGC-7901, AGS, MKN-45 and BGC-823 cells treated with 0μM, 1μM , 10μM and 30μM DIM for 48h.

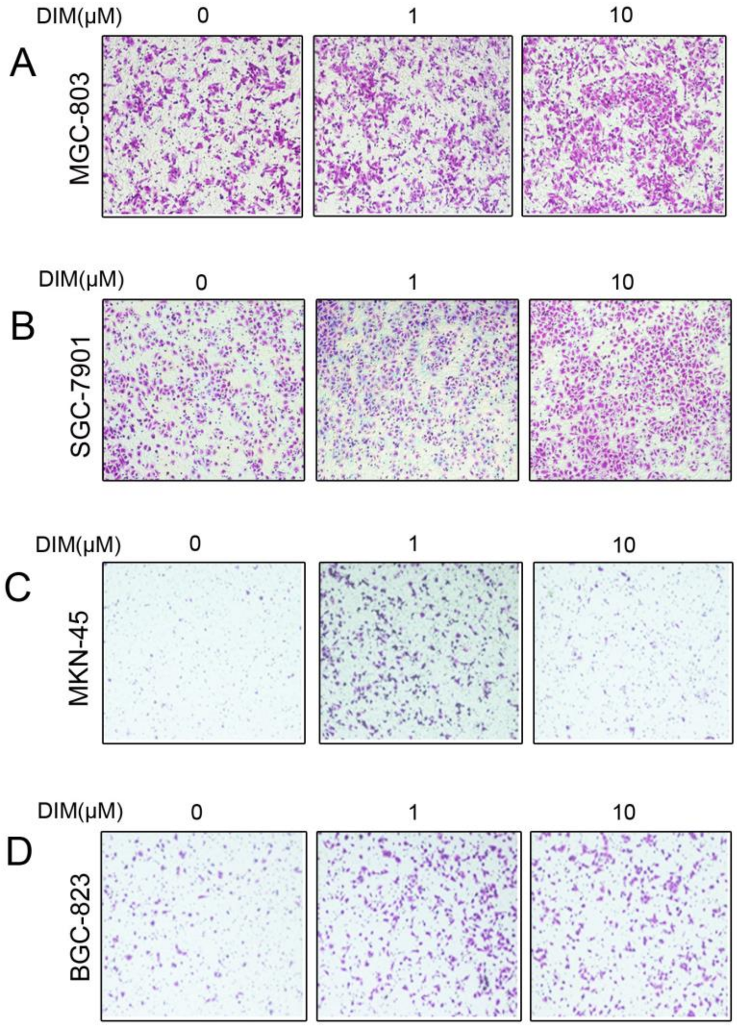

Supplementary Figure 2. **Low level of DIM promoted gastric cancer cells migration**

(A, B, C, D) The migratory ability of MGC-803, SGC-7901, MKN-45 and BGC-823 cells treated with 0μM, 1μM and 10μM DIM for 48h was evaluated by transwell migration assay. Original magnification, 100X.

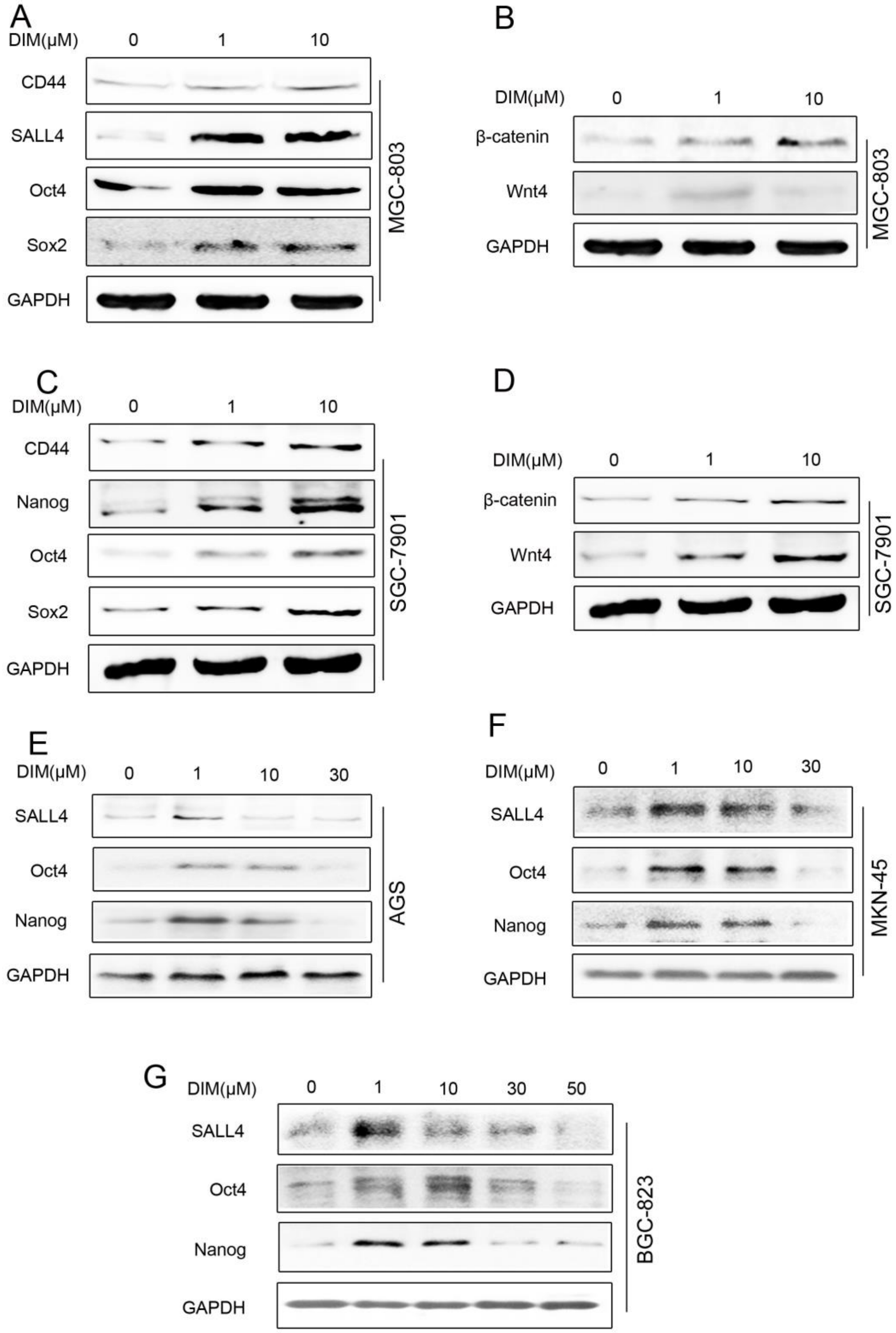

Supplementary Figure 3. **Low level of DIM enhanced gastric cancer cells stemness through Wnt4/β-catenin pathway**

(A, C, E, F, G) Western blot for the expression of stemness proteins in MGC-803, SGC-7901, AGS, MKN-45 and BGC-823 cells treated with 0μM, 1μM and 10μM DIM for 48h. (B, D) Western blot for the expression of Wnt4 and β-catenin proteins in MGC-803 and SGC-7901 cells treated with 0μM, 1μM and 10μM DIM for 48h.

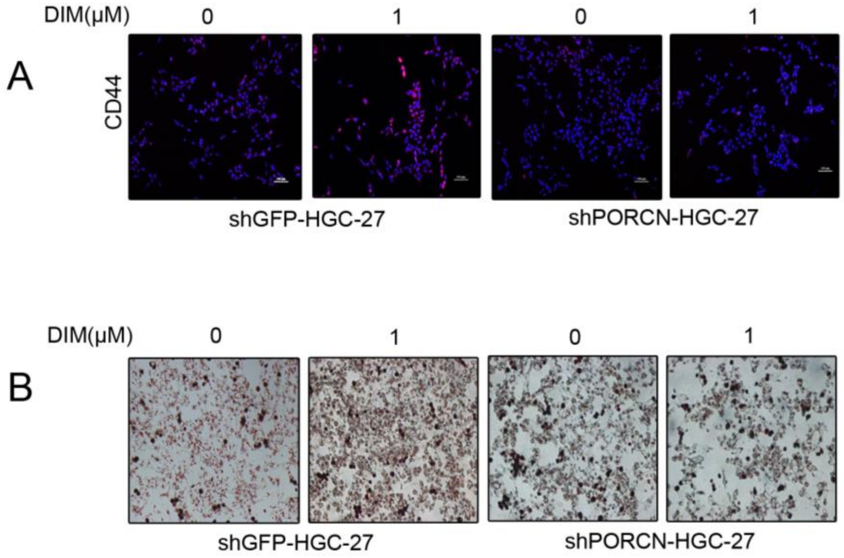

Supplementary Figure 4. **Low level of DIM enhanced stemness of gastric cancer cells through inducing Wnt autocrine to activate β-catenin pathway**

(A) Immunofluorescent staining of CD44 in shGFP-HGC-27 and shPORCN-HGC-27 cells treated with 0μM or 1μM DIM for 48h. Original magnification, 200X. (B) Adipogenic differentiation of shGFP-HGC-27 and shPORCN-HGC-27 cells after treatment with 0μM and 1μM DIM for 48h. Original magnification, 200X.

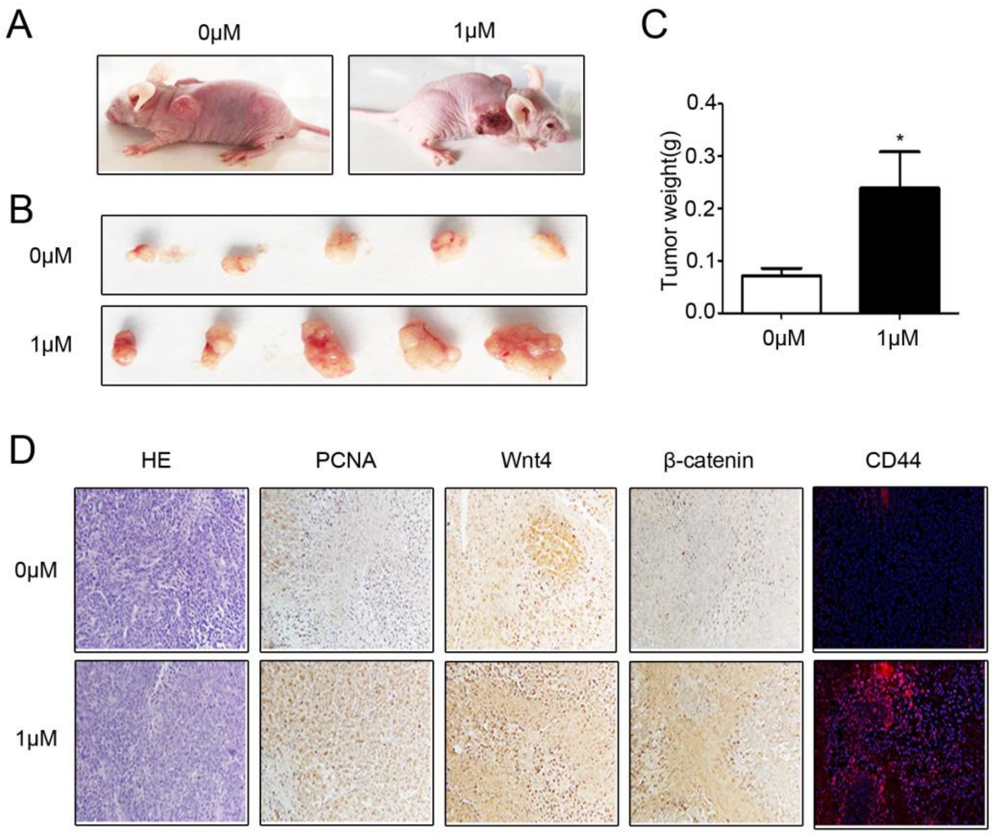

Supplementary Figure 5. **Low level of DIM promoted gastric cancer growth *in vivo*** (A) Representative images of tumor-bearing mice. (B) The photograph of excised tumors at 30 days post inoculation. (C) Tumor weight was evaluated in mice transplanted with MGC-803 cells that were treated with 0μM or 1μM DIM for 48h (n=5, *P<0.05). (D) The subcutaneous tumors derived from MGC-803 cells treated with 0μM (upper) and 1μM DIM (lower) was subjected to H&E staining, immunohistochemical staining of PCNA, Wnt4 and β-catenin, immunofluorescent analyses of CD44 expression. Original magnification, 200X.

Supplementary Table 1. Primer Sequences

mRNA	Primer	Sequences(5'-3')	Annealing temperature
Human-Oct4	Forward primer	TTGAGGCTCTGCAGCTTAG	60℃
	Reverse primer	GCCGGTACAGAACCACAC	
Human-SALL4	Forward primer	TCGATGGCCAACTTCCTTC	62℃
	Reverse primer	GAGCGGACTCACACTGGAGA	
Human-Sox2	Forward primer	ACACCAATCCCATCCACACT	60℃
	Reverse primer	GCAAACTTCCTGCAAAGCTC	
Human-WNT1	Forward primer	GATCGTCAACCGAGGCTGTC	64℃
	Reverse primer	CGTGCAGGATTCGATGGAAC	
Human-WNT2	Forward primer	AGCTGGCAGGAAGGCTGTAA	63℃
	Reverse primer	CAGCCAGCATGTCCTGAGAG	
Human-WNT3	Forward primer	GGCGCCTCTTCTAATGGA	60℃
	Reverse primer	AGAAGCGCAGTTGCTTGG	
Human-WNT3a	Forward primer	GGCATGATCTCCACGTAGTT	63℃
	Reverse primer	TACTCCTCTGCAGCCTGAAG	
Human-WNT4	Forward primer	GCGAGCAACTGGCTGTACCT	64℃
	Reverse primer	AGGTTCCGCTTGCACATCTG	
Human-WNT5a	Forward primer	CTCGCCATGAAGAAGTCCA	59℃
	Reverse primer	TACCTAGCGACCACCAAGAA	
Human-WNT6	Forward primer	GACGCATCCTGCAACAGGAC	65℃
	Reverse primer	AGCAGCTCGCCCATAGAACA	
Human-WNT7b	Forward primer	CGAAGCGGAACTGGTACTGG	64℃
	Reverse primer	TGAAGCTCGGAGCACTGTCA	
Human-WNT10b	Forward primer	GGCGCCAGGTGGTAACTGAA	66℃
	Reverse primer	GCTCCAGAATTGCGGTTGTG	
Human-WNT11	Forward primer	ACAAGACAGGCAGTGCAACA	61℃
	Reverse primer	ACGTAGCAGCACCAGTGGTA	
Human-PORCN	Forward primer	TGCCATGTCACGGTGTCTAC	61℃
	Reverse primer	TACCTCGTGCTGTTCCTCTG	
Human-Snail	Forward primer	GCGAGCTGCAGGACTCTAAT	60℃
	Reverse primer	GCCTCCAAGGAAGAGACTGA	
Human-N-cadherin	Forward primer	AGTCAACTGCAACCGTGTCT	60℃
	Reverse primer	AGTCAACTGCAACCGTGTCT	
Human-β-actin	Forward primer	CACGAAACTACCTTCAACTCC	56℃
	Reverse primer	CATACTCCTGCTTGCTGATC	
